# Migratory singers dynamically overlap the signal space of a breeding warbler community

**DOI:** 10.1002/ece3.11013

**Published:** 2024-02-24

**Authors:** Joanna M. Sblendorio, Maarten J. Vonhof, Sharon A. Gill

**Affiliations:** ^1^ Department of Biological Sciences Western Michigan University Kalamazoo Michigan USA; ^2^ Institute of the Environment and Sustainability Western Michigan University Kalamazoo Michigan USA

**Keywords:** birdsong, migratory species, Parulidae, signal space partitioning, signaling niche, species interactions

## Abstract

Migratory species inhabit many communities along their migratory routes. Across taxa, these species repeatedly move into and out of communities, interacting with each other and locally breeding species and competing for resources and niche space. However, their influence is rarely considered in analyses of ecological processes within the communities they temporarily occupy. Here, we explore the impact of migratory species on a breeding community using the framework of acoustic signal space, a limited resource in which sounds of species within communities co‐exist. Migrating New World warblers (Parulidae, hereafter referred to as migrant species) often sing during refueling stops in areas and at times during which locally breeding warbler species (hereafter breeding species) are singing to establish territories and attract mates. We used eBird data to determine the co‐occurrence of 19 migrant and 11 breeding warbler species across spring migration in SW Michigan, generated a signal space from song recordings of these species, and examined patterns of signaling overlap experienced by breeding species as migrants moved through the community. Migrant species were present for two‐thirds of the breeding season of local species, including periods when breeding species established territories and attracted mates. Signaling niche overlap experienced by individual breeding species was idiosyncratic and varied over time, yet niche overlap between migrant and breeding species occurred more commonly than between breeding species or between migrant species. Nevertheless, the proportion of niche overlap between migrant and breeding warblers was similar to overlap among breeding species. Our findings showed that singing by migrant species overlapped the signals of many breeding species, suggesting that migrants could have unexplored impacts on communication in breeding species, potentially affecting song detection and song evolution. Our study contributes to a growing body of research documenting the impacts of migratory species on communities and ecosystems.

## Graphical abstract

Migratory species inhabit ecological communities for short periods of time, with largely unknown impacts on breeding species and their interactions. Our study explored partitioning of signal space among migratory and locally breeding songbird species, finding that the songs of migrants overlapped those of many breeding species in signal space. Acoustic overlap occurred during critical periods of mate attraction and territorial defense, suggesting that singing by migrants could have important and unknown effects on communication, including signal detection and evolution, in breeding bird communities.

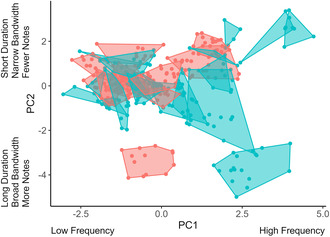

## 1 INTRODUCTION

Migratory species move thousands of kilometers between non‐breeding and breeding areas, entering and exiting multiple ecological communities over relatively short periods of time (Schmaljohann et al., 2022). While temporarily residing in communities during these stopovers, migratory species (hereafter “migrant species”) co‐occur with each other and with locally breeding species (hereafter “breeding species”), yet the extent and impacts of possible interactions among these species remain largely unexplored (Bauer & Hoye, 2014; Schlägel et al., 2020). The presence of migratory species temporarily changes regional species pools (Krishnan, 2019), leading to ecological interactions that vary over time, with likely impacts on species occupying similar niches as migrants (Schlägel et al., 2020). Moreover, as migrants near their own breeding areas, they may enter communities in which many species have initiated breeding activities; as a result, migrants may compete with locally breeding species for resources and niche space during vital life history stages. Understanding impacts of short‐term pulses of migratory species on breeding communities and patterns of niche partitioning may provide critical insight into their influence on ecological systems (Bauer & Hoye, 2014; Schlägel et al., 2020).

A novel approach to exploring ecological impacts of migratory species examines partitioning of signal space in breeding songbird communities during periods of influx by migrant bird species. Within breeding communities, species vocalizing at the same time and place may experience signal masking due to both intraspecific and interspecific overlap in signal space (i.e., a multidimensional area defined by spectral and temporal features of signals; Nelson & Marler, 1990; Schmidt & Balakrishnan, 2015). To minimize acoustic interference, co‐existing species are predicted to inhabit distinct, non‐overlapping regions of signal space, as observed in breeding communities across diverse taxa (birds: e.g., Luther, 2009, frogs: e.g., Chek et al., 2003, insects: e.g., Schmidt et al., 2013, bats: e.g., Kingston et al., 2000, and fish: e.g., Bertucci et al., 2020). In addition to the vocalizations of locally breeding species, migrant bird species often sing when they temporarily reside in a stopover habitat to refuel for the next leg of their journeys (Gahr, 2020; Sblendorio & Gill, 2023). Migrant species use the presence of seasonally breeding species (i.e., both other migrants that breed in an area and year‐round resident species) as cues to habitat quality and food availability in unfamiliar areas (Rodewald & Brittingham, 2002), such that breeding and migrant species commonly co‐occur. Depending on the extent of singing and overlap with breeding species, which is currently unknown, migrants could have important but overlooked influences on the structure of signal space within breeding communities. Given the high probabilities of co‐occurrence, how might singing by migrant species affect patterns of acoustic partitioning and overlap in signal space in breeding bird communities?

The brevity of migration, paired with interspecific differences in both the timing of migration (Helm et al., 2009) and the extent of singing during stopovers (Sblendorio & Gill, 2023), may lead to dynamic patterns of overlap in and competition for signal space. Differences in the timing of arrival at stopovers could stagger and prolong acoustic overlap among migrant and breeding species, such that males of breeding species, singing to defend territories and attract mates, are likely to be confronted with a continuously changing acoustic community. Interspecific differences in timing of arrival could segregate migrant and breeding species (Heim et al., 2018), leading to minimal overlap at any one moment in time or short bursts of interference. Alternatively, more clustered arrival by migrants could lead to high levels of overlap of breeding species, affecting song detection and discrimination by conspecifics. In addition to understanding patterns of acoustic partitioning of signal space, understanding the influence of temporary influxes of migratory singers will help to elucidate additional selective pressure on the songs of breeding species (see also Krishnan, 2019).

In this paper, we explored the signal space generated by the songs of migrant and locally breeding males of New World warblers (Parulidae; hereafter “warblers”). All warbler species in the continental United States (where we conducted our study) are migratory, and at any given latitude, warbler communities vary in composition over time with migrants of various species residing briefly at a stopover site while moving through a region to reach their more northerly breeding sites and co‐occurring with other species that already migrated to their breeding locations. Migrant and breeding warblers are likely to co‐occur and share signal space: migrant warblers join interspecific flocks (Zou et al., 2018), breeding warblers show high species co‐occurrence (Cohen & Satterfield, 2020; Lovette & Hochachka, 2006), and critically, warblers sing during migratory stopovers (e.g. Cox, 1960; Janes, 2017; Morse, 1991; Sblendorio & Gill, 2023; Trautman, 1940).

**FIGURE 1:**
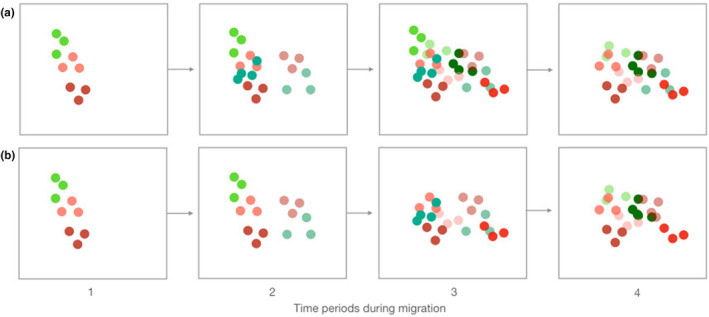
A conceptual diagram illustrating two possible outcomes for partitioning in signal space between migrant and locally breeding warbler species. Panels show four successive time points during the period when migrants are stopping over in the area of breeding species, which are singing to establish territories and attract mates. (a) High overlap in signal space due to high temporal co‐occurrence during migration (migrant species shown in shades of green, *n* = 5; locally breeding species shown in shades of red, *n* = 5). (b) Low overlap in signal space as migrants have staggered migration timing.

Each species in signal space displays an acoustic niche, composed of both signal and perceptual space (Chhaya et al., 2021). Given our focus on warbler song and not receiver dimensions, we introduce the term “signaling niche,” which we define as the area of signal space occupied by the vocalizations of individual species. We examined the hypotheses that the songs of migrant warblers overlap the signal space of breeding warblers and that patterns of signaling niche overlap vary over time depending on the migrant species present in the community. With limited song divergence among sympatrically breeding warblers (Simpson et al., 2021), we expected signaling niche overlap among breeding species. We tested whether overlap between migrant and breeding warblers is similar to patterns of overlap among breeding species and whether the extent of migrant‐breeding overlap varies over time depending on which species were present in the community. If migrant species show low temporal co‐occurrence, few species will be present at any one time and overlap of breeding species' signaling niches by migrants may be low (Figure 1). At the other extreme, if migrant species move through the region more or less simultaneously, then acoustic overlap of breeding species by migrants could be high (Figure 1). Understanding the degree of signaling niche partitioning among migrant and breeding warbler species will provide new dimensions to foundational studies of interspecific competition and niche partitioning among warblers (e.g., Kent & Sherry, 2020; Lack, 1976; Macarthur, 1958; Martin & Martin, 2001). Moreover, our study contributes new perspectives on important and not well understood impacts of migratory species on song evolution and ecological processes in breeding communities (Schlägel et al., 2020).

## 2 METHODS

### 2.1 Song recordings and analysis

We conducted our study of the signal space of migrant and breeding warblers at natural areas in Kalamazoo and Van Buren Counties (42.290 N, 85.586 W), Michigan, U.S.A., between April and July, 2019 and 2020. Migrant warblers move through this region during April–June (Sblendorio & Gill, 2023); thus, we recorded birds when migrants were abundant and also when they had moved out of the region and only breeding species remained in the community. To explore signaling niches and patterns of overlap, we recorded songs of 30 warbler species, categorizing species as local breeders (*n* = 11) or migrants (*n* = 19) based on whether each was confirmed as breeding within Kalamazoo Co. (Chartier et al., 2011; http://www.ebird.org). We minimized recording in areas with high anthropogenic noise levels, which may influence song structure (e.g., Brumm & Slabbekoorn, 2005) and data extraction (Grabarczyk et al., 2018), and used a Marantz PMD561 digital recorder (Kanagawa, Japan) and MKH‐70 Sennheiser long shotgun microphone (Wedemark, Germany) with windscreen (16‐bit, sampling rate of 22.1 kHz). We primarily recorded males between 0500 and 1100 Eastern Daylight Hours (EDT), opportunistically recording them as they were encountered and occasionally targeting uncommon or poorly sampled species by referencing daily eBird (Sullivan et al., 2009) sighting records to find their locations. Recording occurred passively without playback and stopped when males moved out of range (approximately >20 m) or could no longer be followed (mean recording duration: 511 s, range: 54–1879 s, *n* = 329 recordings). Note that we did not consider temporal avoidance by species (see Planqué & Slabbekoorn, 2008), as we primarily recorded one species at a time to ensure high‐quality recordings for analysis.

To avoid resampling individuals, we marked recording locations with GPS. On return visits to sites, we recorded males of locally breeding species if they were located greater than 300 m from previously recorded individuals. We minimized resampling of migrant individuals by separating site visits by at least 5 days, unless a target species was reported or the site was sufficiently large for recording in distinct patches on return visits. In total, we recorded 329 individual males, with a minimum of four individuals and a maximum of 24 individuals per species recorded (mean ± SD number of individuals per species = 11.0 ± 6.0, *n* = 30 species; migrant species = 8.4 ± 5.4, *n* = 19 species; breeding species = 15.4 ± 4.2, *n* = 11 species). We did not observe singing males of two migrant species (orange‐crowned warbler, *Leiothlypis celata*, and Louisiana waterthrush, *Parkesia motacilla*), which are not included in this study. Our fieldwork procedures were approved by Western Michigan University's Institutional Animal Care and Use Committee (IACUC No. 19‐01‐02), and permission to record at study sites was granted by the Southwest Michigan Land Conservancy.

**FIGURE 2:**
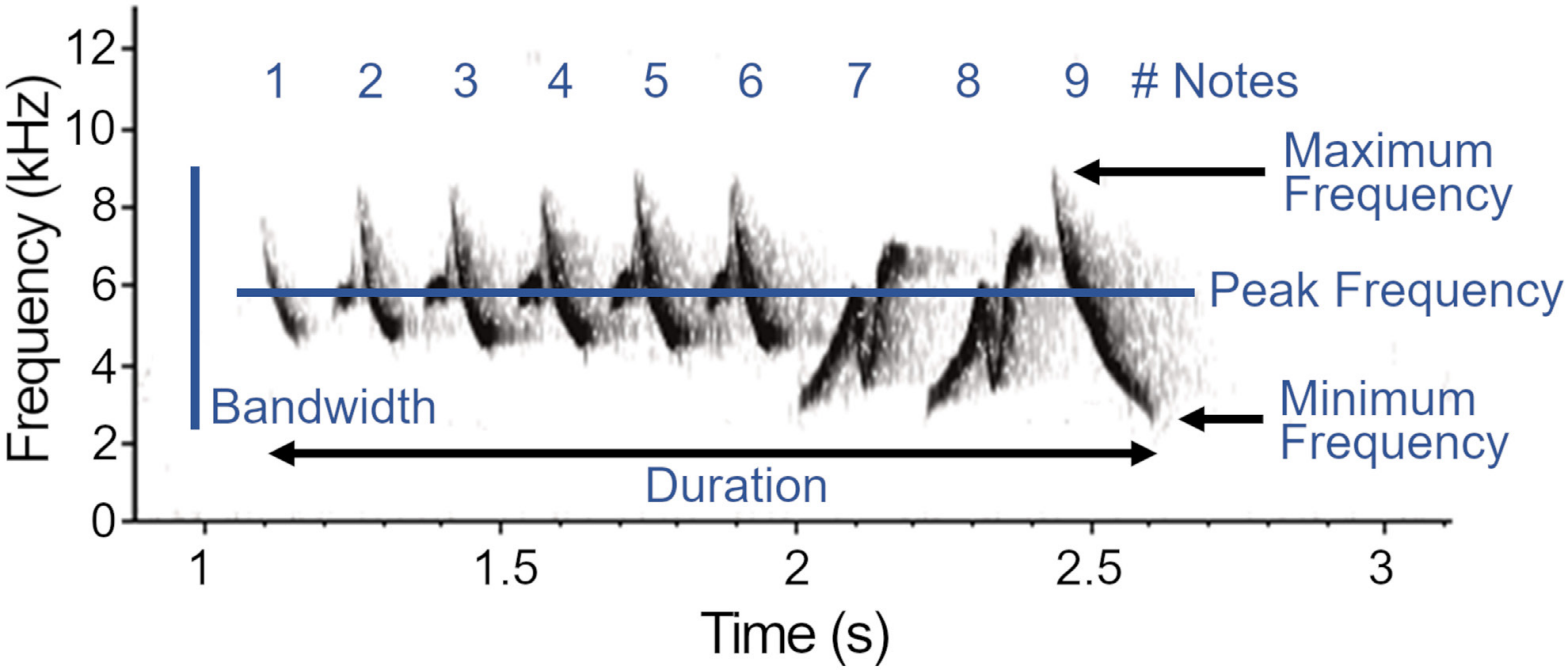
A spectrogram of one song of chestnut‐sided warbler (*Setophaga pensylvanica*), illustrating six song traits measured from the entire songs of 329 individuals across 30 warbler species. Song duration and the frequency characteristics of peak, minimum, and maximum frequency, as well as bandwidth (maximum–minimum frequency) were measured using the automated parameter tool in Avisoft (threshold to extract minimum and maximum frequencies was set at −15 dB relative to peak frequency). The number of notes in a song were manually counted in the spectrogram view.

We analyzed 2746 warbler songs (mean ± SD number of songs per species = 91.5 ± 55.6, *n* = 30 species; migrant species = 67.9 ± 50.8; breeding species = 132.3 ± 38.2) using Avisoft‐SASLab Pro v5.2.13 (Specht, 2002). We manually labeled songs with a cursor on spectrograms (Flat top window, 512 FFT sample length, 93.75% overlap, 0.725 ms time resolution) and omitted songs overlapped by ambient noise or vocalizations of other species. Prior to extraction of song traits, we applied species‐specific band pass filters (Table A1) to remove high‐ and low‐frequency background noise. We developed filters by estimating the frequency range of five randomly selected songs per individual per species and set filters to be a minimum of 0.5 kHz above and below the frequency range. After filtering, we used the automated parameter measurement tool (threshold −15 dB on the power spectrum) to extract six features characterizing the whole song (Figure 2): overall duration (s), number of notes, peak frequency (frequency of the highest amplitude, Hz), minimum frequency (Hz), maximum frequency (Hz), and bandwidth (range of frequencies, Hz). We adopt metrics that are repeatable across species with different song structures, following previous studies of signal space and acoustic partitioning (e.g., Allen‐Ankins & Schwarzkopf, 2022; Planqué & Slabbekoorn, 2008; Tobias et al., 2014; but see, e.g., Chitnis et al., 2020 for analysis of notes), as well as song evolution in birds generally (e.g., Arato & Fitch, 2021) and warblers specifically (Simpson et al., 2021).

### 2.2 Composition of the warbler community over time

To estimate the composition of the migrant and breeding warbler community over time, we used eBird (Sullivan et al., 2009) data. We extracted dates when warbler species first appeared at our sites in SW Michigan and when migrants left the region, thereby visualizing the timing of stopovers for each species. We used 10 years of eBird observations submitted between 2011 and 2020 and downloaded a subset of the eBird basic dataset (version ebd‐relNov‐2020; available at ebird.org/data/download) reduced to checklists from Kalamazoo Co. Following best‐use practices (Johnston et al., 2021), we filtered the dataset using the R package *auk* (Strimas‐Mackey et al., 2018), which standardized the data by accounting for variation in detectability among checklists. We retained complete checklists with either stationary or traveling protocols submitted between April 1 and June 15 for all years and eliminated checklists with more than 10 observers, a duration greater than 5 h, and a distance over 5 km (Johnston et al., 2021). We further filtered the data to remove duplicate lists and observations above the species level (Johnston et al., 2021). After filters were executed, 7282 eBird checklists remained for analysis.

Using the filtered dataset, we calculated frequency of occurrence (“occurrence” hereafter) during migration for each of the 30 warbler species in Kalamazoo Co. between 2011 and 2020. eBird (Sullivan et al., 2009) defines occurrence as the percentage of checklists reporting a species on a given date. A species was considered “present” in the acoustic community when its occurrence was 1.5% or greater. This threshold allowed us to include all species that we observed singing during migration in the region, even those that were uncommon and did not exceed an occurrence of 2% (e.g., golden‐winged warbler). Moreover, the areas of species' signaling niches reflected natural abundances (see Section 3), which means the inclusion of uncommon species did not lead to overestimates of overlap within the community. This cutoff value also excluded the trickle of individuals at the beginning and/or end of a species' migratory period and therefore only included species when they were likely to be abundant. From these data, we identified April 5 as the earliest date across years that any migrant species was observed in Kalamazoo Co. and June 6 as the earliest date that the community included only locally breeding species. To reduce possible temporal autocorrelation, we averaged occurrence values from 10 years of checklists across 3‐day intervals between April 5 and June 6 (Julian days 96 to 158, respectively). Using average occurrence values, we then re‐constructed species composition of the warbler community at 21 time points during migration, from which we extracted data on co‐occurrence among warblers.

### 2.3 Visualization of signal space

All analyses were performed using R (Version 4.0.2) (R Core Team, 2020) except as otherwise specified. We visualized the maximum potential for overlap by generating a signal space that included all species and then focused on patterns of acoustic overlap resulting from warbler co‐occurrence at the 21 time points. To visualize signal space, and calculate niche areas and pairwise overlap of niches, we first averaged the individual song parameters measured per male (mean ± SD = 8.3 ± 2.4 songs per male) and then performed a principal component analysis (PCA) on the correlation matrix of the average song characteristics per male (*n* = 329 males) of the 30 warbler species. The PCA yielded two principal components (PCs) with eigenvalues greater than one and these together explained 79% of total variation (Table A2). PC1 explained 41.2% of variation among species and heavily loaded variables reflecting song frequency (peak frequency, maximum frequency, and minimum frequency). PC2 explained 37.5% of variation and heavily loaded variables reflecting the temporal characteristics of songs (song duration and number of notes) and song bandwidth. These two PCs defined the axes of a two‐dimensional (2‐D) signal space within which each of the 30 species' signaling niches were located and measured. We used the R package *hypervolume* (Blonder, 2018) within the 2‐D signal space to generate convex hulls, which represented species' signaling niches and from which we calculated niche area for each species.

**FIGURE A1:**
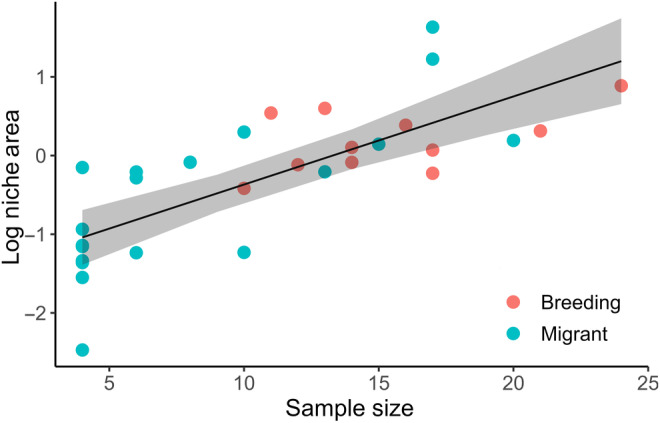
A linear regression modeling the effect of sample size on log‐transformed signaling niche area, showing estimates for the model fit (line) plus upper and lower 95% confidence intervals (shading; *R* ^2^ = .56, *F*_1,28_ = 36.24, *p* < .0001). Solid dots represent migrant (blue, *n* = 19) and breeding (red, *n* = 11) species.

Sample size may influence the calculation of total niche area of a given species (Brandl & Bellwood, 2014; González et al., 2017). For example, individuals with extreme traits can expand total niche area and the probability of sampling individuals inhabiting extreme positions in trait space increases with increasing sample size (Brandl & Bellwood, 2014). We calculated signaling niche areas based on samples of 4–24 individuals per species and assessed the impact of sample size on niche areas in two ways. First, we ran a linear regression to test the influence of the number of individuals recorded per species on the species' log‐transformed niche areas and tested whether the impact of sample size differed between migrant and breeding warblers. We included the number of individuals recorded per species (continuous), migratory status (categorical with two levels: migrant, breeding), and the interaction between these terms. There was evidence for multicollinearity in the model as no coefficient estimates were significant yet the model had a high *R* ^2^ (*R* ^2^ = .62). We also calculated variance inflation factors (VIFs) for each predictor in the model and found that migratory status exceeded the cut‐off value of 10 (migratory status VIF = 11.05), suggesting an unacceptable level of multicollinearity (O'Brien, 2007). Therefore, we re‐ran the linear model with only the number of individuals recorded per species as a predictor and found that the number of individuals recorded per species was positively related to log‐transformed niche area (*R* ^2^ = .56, *F*_1,28_ = 36.24, *p* < .0001; Figure A1). These results suggest that subsequent analysis could be biased by sample size differences across species.

Sample size may also reflect observed species abundance within a community (González et al., 2017). If so, then correcting for sample size across species may inflate niche areas for less abundant species, thereby leading to higher estimates of species overlaps in niche space (González et al., 2017). In our second approach, we explored whether we sampled species in proportion to their natural abundances in our study region by documenting presence/absence of species at each recording site on the days we visited the site and calculating a total count of encounters for each species. We tested whether the number of days a species was encountered in the field predicted the number of individuals recorded using a Poisson generalized linear model (GLM) with a log‐link function. Predictors were the number of encounters per species (continuous), migratory status (categorical with two levels: migrant, breeding), and the interaction between these terms. Validation of the model did not indicate overdispersion.

**FIGURE A2:**
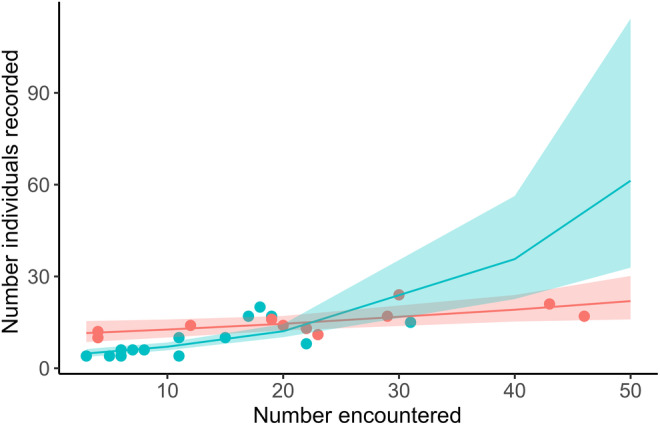
A Poisson generalized linear model testing whether species were sampled relative to their natural abundances. The predictors were number of encounters per species (continuous), migratory status (categorical with two levels: breeding, migrant), and the interaction term (number of encounters × migratory status; *F*_1,26_ = 14.21, *p* < .0001), with number of individuals recorded per species as the response variable. This shows estimates for the model fit (lines) with migratory species in blue, breeding species in red, and upper and lower 95% confidence intervals (shading). Solid dots represent migrant (blue, *n* = 19) and breeding (red, *n* = 11) species.

Overall, species were sampled in proportion to their natural abundances, as the number of species encounters in the field predicted the number of individuals recorded (number encountered term: *F*_1,26_ = 51.26, *p* < .0001). However, a significant interaction between number of encounters and migratory status existed (number of encounters x migratory status term: *F*_1,26_ = 14.21, *p* < .0001; Figure A2): migrant species were sampled in proportion to the number of encounters, whereas this relationship did not hold for breeding species. The latter result reflected our ability to record breeding species over a longer period of time and minimize sample size differences across these species. Thus, although sample size was positively related to total niche area, we sampled species in proportion to their natural abundances in SW Michigan and therefore did not correct for sample size in subsequent analyses.

### 2.4 Community patterns of niche overlap

To examine patterns of niche overlap by migrants on breeding species through time, we calculated two measures, area of overlap and number of overlaps between signaling niches, between all species pairs in our dataset. We considered all nonzero overlaps between signaling niches in these calculations regardless of area of overlap (that is, we did not set a minimum threshold of area of overlap to be counted). These measures of overlap were quantified using the overall 2‐D signal space, assuming the potential for all species in our dataset to co‐occur at the same place and time, as well as for each of the reconstructed communities (*n* = 21), which reflect pairwise species coexistence during each 3‐day interval during the migratory period. In each case, we coded each species combination as migrant and breeding pairs, breeding and breeding pairs, and migrant and migrant pairs to ask whether patterns of overlap differed across pair types.

To understand the extent of niche overlap experienced by breeding species, we used *hypervolume* to calculate the proportion of signaling niches of each breeding species that was overlapped by migrants, other breeding species, and in total. For each of these categories, we calculated the maximum proportion overlap possible, assuming the entire community was present simultaneously (Table 1), as well as the proportion overlap for each 3‐day interval during the migratory period to examine overlap through time. We used a binomial generalized linear mixed model (GLMM) with a logit‐link function and species as a random effect to test whether overlap type (breeding, migrant), day of year, and their interaction influenced the proportion of niche overlap experienced by breeding species. Prior to analysis, day of year was centered to a mean of 0 and scaled to a standard deviation of 1, as recommended by Schielzeth (2010), to evaluate multicollinearity and improve GLMM interpretability. We used model selection to determine whether models required a quadratic or cubic term to account for non‐linear patterns of migration (migrant species arrive in small numbers, become abundant, and then drop to zero once they leave the area). Although model selection revealed that the quadratic model was the best fit (Table A3), overdispersion was significant (dispersion parameter = 16,070) and persisted when we fit the model using alternative distributions (quasibinomial, beta‐binomial). Therefore, we selected the model with the cubic term for analysis; validation of the model did not indicate overdispersion (dispersion parameter = 2.56) and the cubic and quadratic models did not differ in deviance (Table A3). Finally, to determine whether breeding species experienced greater overlap from migrants or from other breeding species at the peak of migration, we randomly selected one of three 3‐day periods when the community had the greatest number of species and performed a paired samples *t*‐test to compare the proportion overlap of breeding species' niches by each group (breeding, migrant).

**TABLE 1:** The 11 breeding and 19 migratory species included in this study.

Species			Status	Number of individuals	Niche area	Overlap		
Common name	Scientific name	Code				Total	Migrant	Breeding
American Redstart	*Setophaga ruticilla*	AMRE	Breeding	24	2.4280	0.5208	0.4093	0.1813
Bay‐breasted Warbler	*Setophaga castanea*	BBWA	Migrant	4	0.3191	0.0106	0.0000	0.0106
Black‐and‐white Warbler	*Mniotilta varia*	BAWW	Migrant	8	0.9182	0.9489	0.6966	0.6294
Blackburnian Warbler	*Setophaga fusca*	BLBW	Migrant	17	5.1158	0.2666	0.2247	0.1017
Blackpoll Warbler	*Setophaga striata*	BLPW	Migrant	6	0.7533	0.1545	0.1545	0.0000
Black‐throated Blue Warbler	*Setophaga caerulescens*	BTBW	Migrant	4	0.2627	0.7903	0.6751	0.6862
Black‐throated Green Warbler	*Setophaga virens*	BTNW	Breeding	11	1.7176	0.5335	0.5050	0.1678
Blue‐winged Warbler	*Vermivora cyanoptera*	BWWA	Breeding	17	0.7984	0.4874	0.1633	0.4835
Canada Warbler	*Cardellina canadensis*	CAWA	Migrant	6	0.2908	0.9717	0.9604	0.9200
Cape May Warbler	*Setophaga tigrina*	CMWA	Migrant	4	0.8590	0.1355	0.1355	0.0000
Cerulean Warbler	*Setophaga cerulea*	CERW	Breeding	12	0.8898	0.9700	0.7161	0.9418
Chestnut‐sided Warbler	*Setophaga pensylvanica*	CSWA	Breeding	16	1.4707	0.9533	0.7211	0.9160
Common Yellowthroat	*Geothlypis trichas*	COYE	Breeding	17	1.0724	0.8552	0.5531	0.7103
Connecticut Warbler	*Oporornis agilis*	CONW	Migrant	4	0.3918	0.0098	0.0098	0.0000
Golden‐winged Warbler	*Vermivora chrysoptera*	GWWA	Migrant	4	0.2123	0.9522	0.1281	0.9485
Hooded Warbler	*Setophaga citrina*	HOWA	Breeding	14	0.9171	0.4225	0.2619	0.2744
Magnolia Warbler	*Setophaga magnolia*	MAWA	Migrant	20	1.2118	0.8684	0.1629	0.8368
Mourning Warbler	*Geothlypis philadelphia*	MOWA	Migrant	4	0.2566	0.9418	0.0000	0.9418
Nashville Warbler	*Leiothlypis ruficapilla*	NAWA	Migrant	15	1.1557	0.7959	0.7934	0.0255
Northern Parula	*Setophaga americana*	NOPA	Migrant	10	1.3480	0.7852	0.4255	0.4414
Northern Waterthrush	*Parkesia noveboracensis*	NOWA	Migrant	10	0.2920	0.7037	0.3882	0.4020
Ovenbird	*Seiurus aurocapilla*	OVEN	Breeding	13	1.8217	0.0000	0.0000	0.0000
Palm Warbler	*Setophaga palmarum*	PAWA	Migrant	4	0.3148	0.9732	0.8875	0.9708
Pine Warbler	*Setophaga pinus*	PIWA	Breeding	14	1.1094	0.9663	0.5508	0.8728
Prairie Warbler	*Setophaga discolor*	PRAW	Migrant	4	0.0845	0.9573	0.5182	0.9527
Prothonotary Warbler	*Protonotaria cotreat*	PROW	Breeding	10	0.6590	0.9274	0.9156	0.3032
Tennessee Warbler	*Leiothlypis peregrina*	TEWA	Migrant	17	3.4065	0.0097	0.0097	0.0000
Wilson's Warbler	*Cardellina perilla*	WIWA	Migrant	6	0.8118	0.8827	0.6616	0.8576
Yellow Warbler	*Setophaga petechia*	YEWA	Breeding	21	1.3677	0.4812	0.4153	0.3469
Yellow‐rumped Warbler	*Setophaga coronata*	YRWA	Migrant	13	0.8131	0.6612	0.1331	0.5511

*Note*: Signaling niche areas are in PCA units. Niche overlap values represent proportions of overlap in two‐dimensional signal space and include total overlap experienced (migrant and breeding overlap), overlap from migrant species, and overlap from breeding species.

Finally, we considered whether the number of overlaps of breeding species by migrant species differed from the number by breeding species. We used a Poisson generalized linear model (GLM) with a log‐link function to test whether pair type (migrant‐breeding, breeding‐breeding, migrant‐migrant), standardized day of year, and their interaction influenced the number of signaling niche overlaps between all species pairs. We used model selection to assess whether the model required a quadratic or cubic term to account for migration patterns that were non‐linear. Model selection revealed that the model containing the cubic term was the best fit (Table A4). We verified model assumptions by plotting residuals versus fitted values and by testing for overdispersion of Pearson residuals using a chi‐square test.

### 2.5 Null model test for dispersion of signaling niches

To examine the pattern of dispersion of signaling niches in signal space, we used a null model test following the methods of Chitnis et al. (2020) in MATLAB (Mathworks, Inc., Natick, MA). First, we calculated pairwise Euclidean distances between the signaling niche centroids of all species in our two‐dimensional principal component (PC) space, yielding a mean interspecific distance for the community. Next, we generated independent random distributions along each PC axis that covered the same range of values as our observed dataset. Within this PC space, we randomly sampled each PC axis to construct 10,000 null communities (each with the same number of species and number of individual males per species as our dataset) and calculated the mean interspecific Euclidean distance of each null community. Finally, we generated a *Z* score by comparing the observed mean interspecific distance to the randomized distribution. This analysis tests whether the community exhibits overdispersion in signal space, where larger interspecific distances between signaling niches than expected by chance (indicated by significantly positive *Z* scores; Chitnis et al., 2020) suggest a partitioned signal space. We repeated this analysis for each species pair (*n* = 435 pairs) in our community to determine the proportion of pairs with signaling niches that were either more distant or closer together than expected by chance.

### 2.6 Phylogenetic relatedness and pairwise proximity in signal space

To determine phylogenetic relatedness among warbler species, we downloaded distributions of phylogenetic trees from http://www.birdtree.org (Jetz et al., 2012). We sampled 10,000 “stage 1” trees (i.e., composed of species with genetic data) built from the Hackett family backbone (Hackett et al., 2008) and pruned to include the 30 warbler species in the study. We used TreeAnnotator v1.10.4 (Drummond et al., 2012) to identify a maximum clade credibility (MCC) tree from our sample phylogenies and inferred branch lengths using median node heights and 10% burn‐in. From this tree, we extracted phylogenetic distances for all species pairs.

To map the distances between signaling niches within 2‐D signal space, we used *hypervolume* to calculate species' niche centroids and Euclidean distances between the centroids of all species pairs. We ran a linear model to test if pair type (breeding‐migrant, breeding‐breeding, migrant‐migrant), phylogenetic distance between species pairs, and their interaction predicted log‐transformed Euclidean distance between species' signaling niches in signal space. Multiple comparisons were tested using a post‐hoc Tukey's test (*α* = .05).

## 3 RESULTS

### 3.1 Overall patterns of signaling niche overlap

**FIGURE 3:**
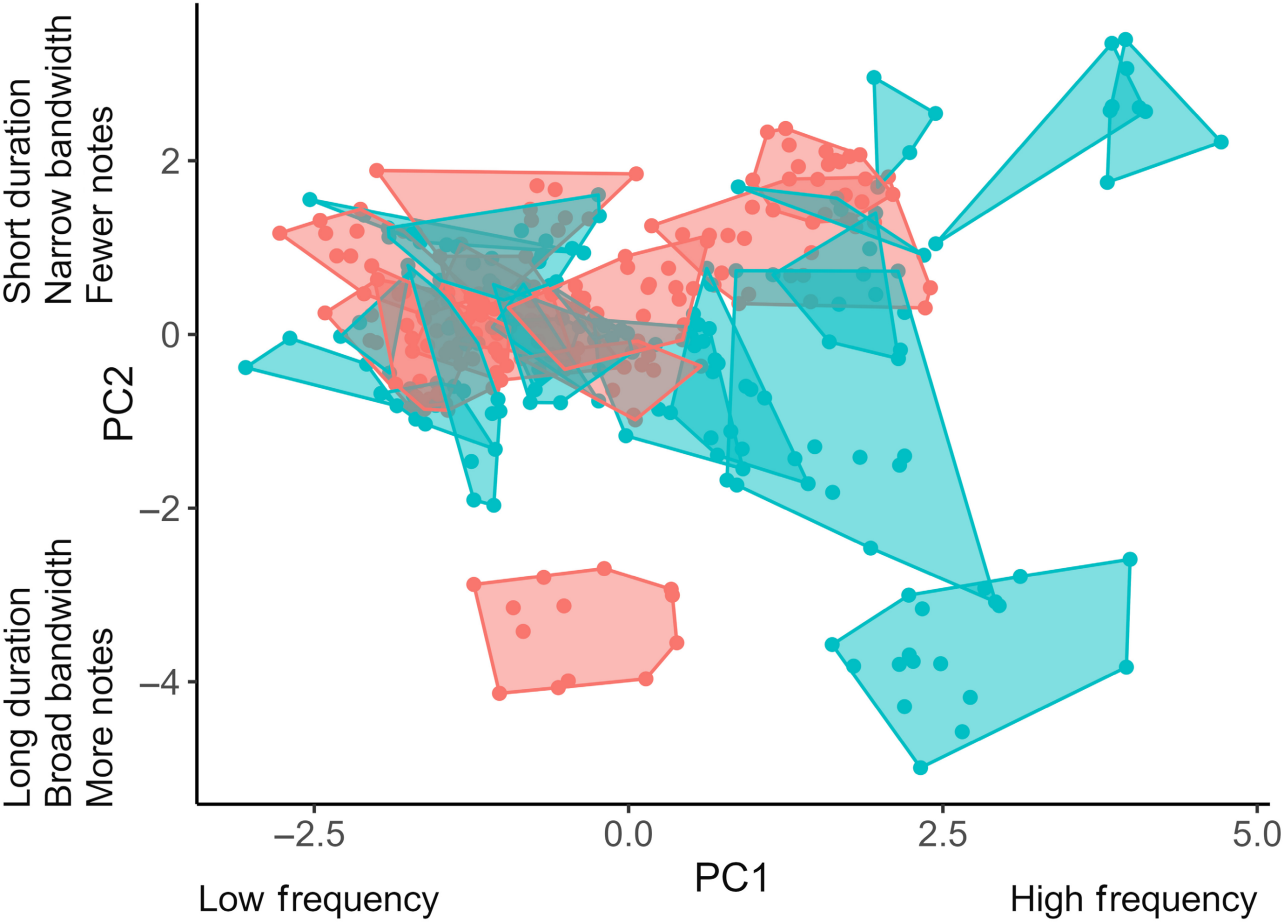
A two‐dimensional signal space of the acoustic community consisting of locally breeding and migrant warblers. The shaded convex hulls represent signaling niches of breeding (*n* = 11; red) and migrant (*n* = 19; blue) species, based on mean song traits per individual (*n* = 329 males, represented by the solid dots). PC1 represents song frequency (minimum, maximum, and peak frequencies) and PC2 reflects song duration, song bandwidth, and number of notes.

Signaling niches of migrant and breeding species varied in area and overlapped to varying degrees (Table 1). Together, migrant and breeding warbler species (*n* = 30) formed a community signal space covering an area of 47.09 units (Figure 3). Areas of signaling niches varied across species from 0.08 to 5.12 units (Table 1, Figure 3; mean niche area ± SD = 1.10 ± 1.04). Signaling niche areas of breeding species ranged from 0.66 to 2.43 units (mean niche area ± SD = 1.30 ± 0.53, *n* = 11) and those of migrant species from 0.08 to 5.12 units (mean niche area ± SD = 0.99 ± 1.25, *n* = 19). Within the community signal space of 30 species, a total of 435 pairwise overlaps of signaling niches were possible, including 209 potential overlaps between migrant‐breeding pairs, 55 between breeding pairs, and 171 between migrant pairs. However, only 84 species pairs (19.3%) overlapped in signal space (Figure 3). Of these 84 overlaps, 46 (54.8%) were migrant‐breeding species pairs, 20 (23.8%) occurred between breeding species, and 18 (21.4%) were between migrants, making migrant‐breeding overlaps the most common by a factor of two (chi‐square goodness‐of‐fit test, *χ* ^2^ = 17.43, *p* < .0002).

The total extent of signaling niche overlap of individual species by one or more others was highly variable (Table 1). Estimates of niche overlap experienced by individual species varied from zero, indicating no overlap by other species, to almost complete overlap (0.97; mean ± SD total proportion niche overlap = 0.63 ± 0.35, *n* = 30). Whereas only one species experienced no acoustic overlap within this community, the signaling niches of five breeding and 11 migrant species were overlapped by those of other warblers by more than 75% (Table 1).

### 3.2 Signaling niche overlap of breeding species by migrants

**FIGURE 4:**
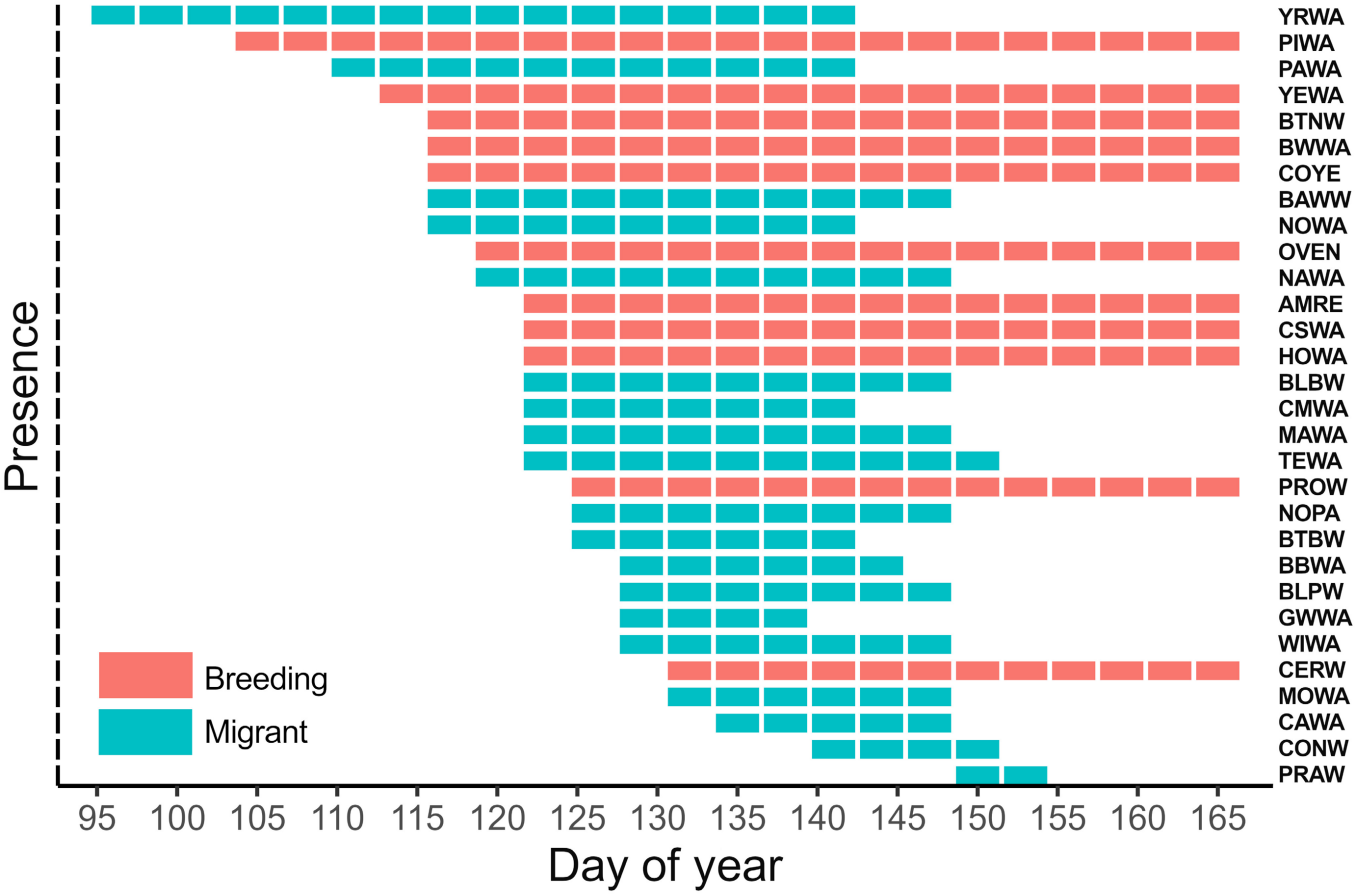
Presence of breeding and migrant warblers within the acoustic community during successive 3‐day periods across the migratory period. A species is considered “present” in the acoustic community when its average frequency of occurrence for the 3‐day period was 1.5% or greater. The consistent end of this figure is an arbitrary cutoff date when only breeding species remained in the community. Refer to Table 1 for species codes.

Migrant warblers were present for extended periods of time during the breeding season of species that breed in SW Michigan (Figure 4). For 51 days of the breeding season (between ~April 14 and June 3), at least one and up to 17 migrant species simultaneously co‐occurred with breeding species. The maximum co‐occurring warbler community consisted of 28 out of 30 species and occurred over a 9‐day period (~May 15–23); this period had the greatest potential overlap within signal space (Figure 4).

**FIGURE A3:**
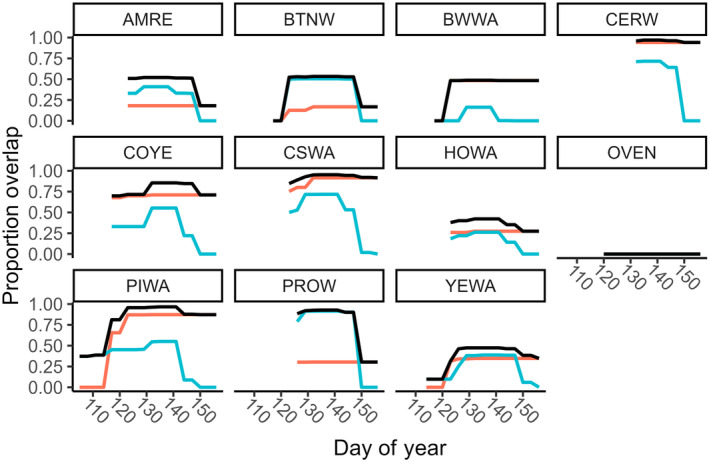
The proportion of niche overlap experienced by locally breeding warblers (*n* = 11) from migrants (blue), other locally breeding species (red), and in total (black) during the migratory period. Refer to Table 1 for species codes.

The proportion of niche overlap experienced by individual breeding species from migrants, other breeding species, and in total varied across species and through time (Table 1, Figure A3). Although mean niche overlap for breeding species (*n* = 11) by migrants and by other breeding species was similar (mean proportion niche overlap ± SD by migrants = 0.47 ± 0.26, range: 0–0.92; by breeding species = 0.47 ± 0.33, range 0–0.94), individual species varied in patterns of overlap by migrants versus breeders. For example, prothonotary warblers (*Protonotaria citrea*) experienced higher niche overlap from migrant compared to breeding species (0.92 and 0.30, respectively; Table 1), whereas cerulean warblers (*Setophaga cerulea*) experienced greater niche overlap by breeding species compared to migrants, although overlap by both types was high (0.94 and 0.72, respectively; Table 1). Ovenbird (*Seiurus aurocapilla*) was the only species whose niche was not overlapped by migrants or breeding species. In total, combined niche overlap by migrants and other breeding species was variable (mean total proportion niche overlap ± SD = 0.65 ± 0.31, range: 0–0.97) and exceeded 0.92 for four breeding species (Table 1). This high level of total overlap of these four breeding species' niches was sustained for at least 21 days of migration (Figure A3).

**FIGURE 5:**
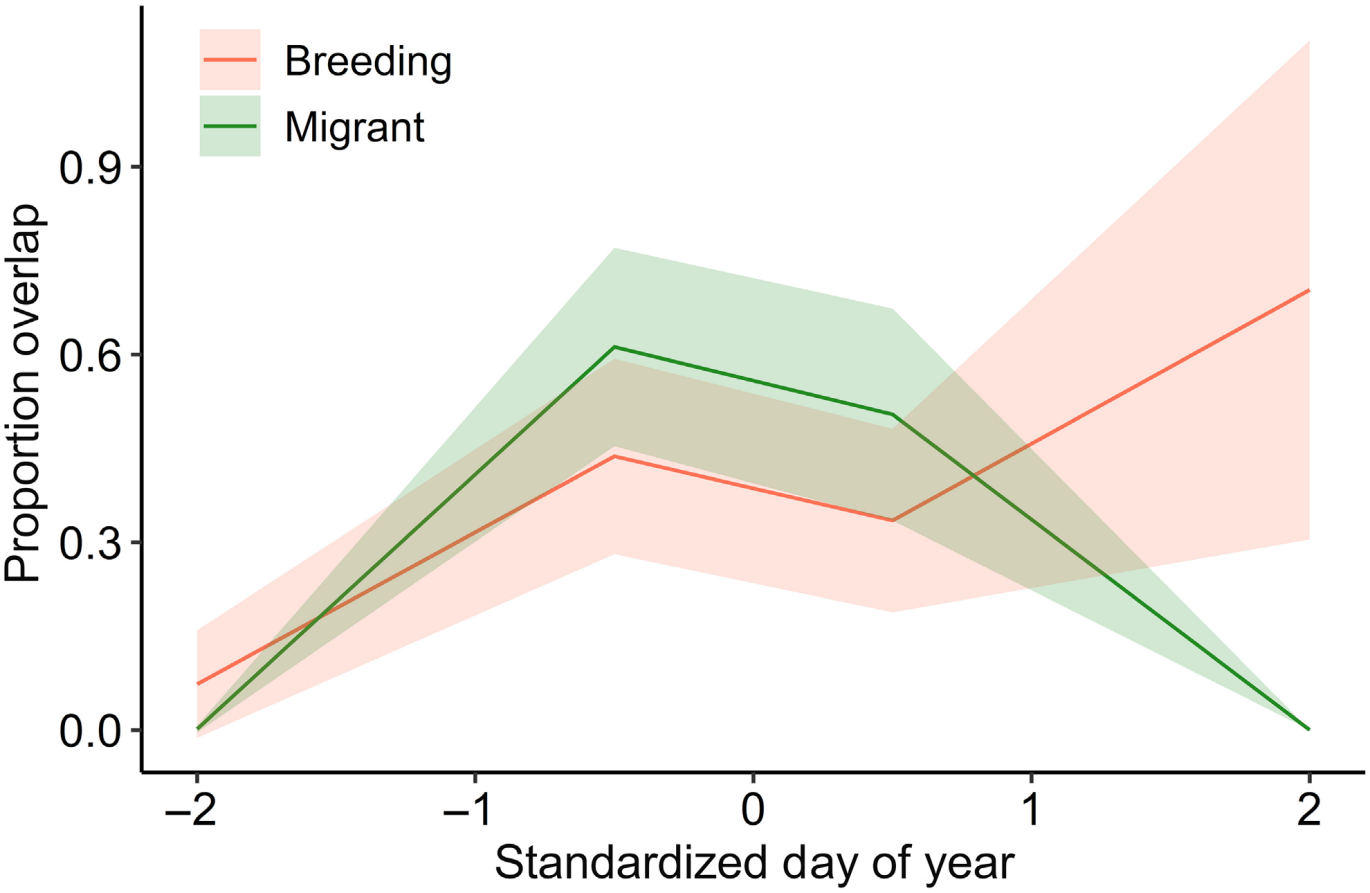
The proportion overlap of breeding species' signaling niches by migrants and other locally breeding species varied over time. The figure displays estimates from a binomial generalized linear mixed model for model fit (solid line) and standard error (shading), with standardized day of year on the *x*‐axis. There was a significant interaction between overlap type (breeding, migrant) and the second‐order cubic polynomial of day of year (interaction term: Wald *χ* ^2^ = 18.25 on 3 df, *p* < .001; quadratic term: *z* = −3.4, *p* < .001).

**FIGURE A4:**
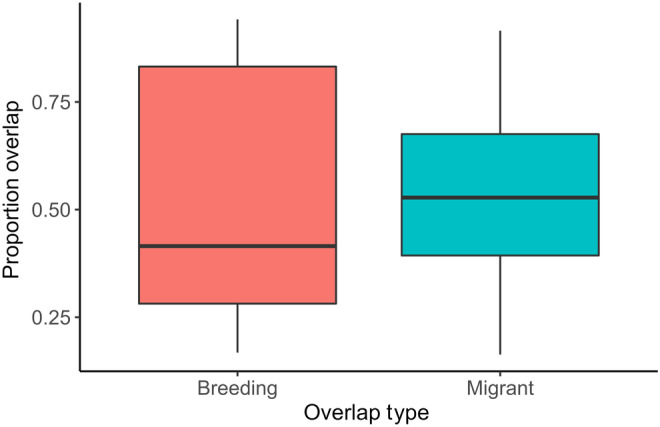
Breeding species experienced a similar proportion of niche overlap from other breeding species (red) and migrants (blue) at the peak of migration (Julian days 135–137) (paired sample *t*‐test: *t*_9_ = 0.017, *p* = .987).

Proportion of overlap of the signaling niches of breeding species was similar between overlap types (breeding, migrant), but varied temporally. There was a significant interaction between overlap type and the second‐order cubic polynomial of date (GLMM, interaction term: Wald *χ* ^2^ = 18.25 on 3 df, *p* < .001; quadratic term: *z* = −3.4, *p* < .001; Table A5). From early to peak migration, breeding species experienced a similar proportion of niche overlap by migrant species as they did from other breeding species (Figure 5). Then, overlap by migrants decreased as they left the area, while overlap from other breeding species was constant once the entire breeding community arrived (Figure 5). Additionally, during the peak of migration (Julian days 135–137), the proportion overlap of breeding species' niches by migrants and other breeding species was similar (paired sample *t*‐test: *t*_9_ = 0.017, *p* = .987; Figure A4).

**FIGURE 6:**
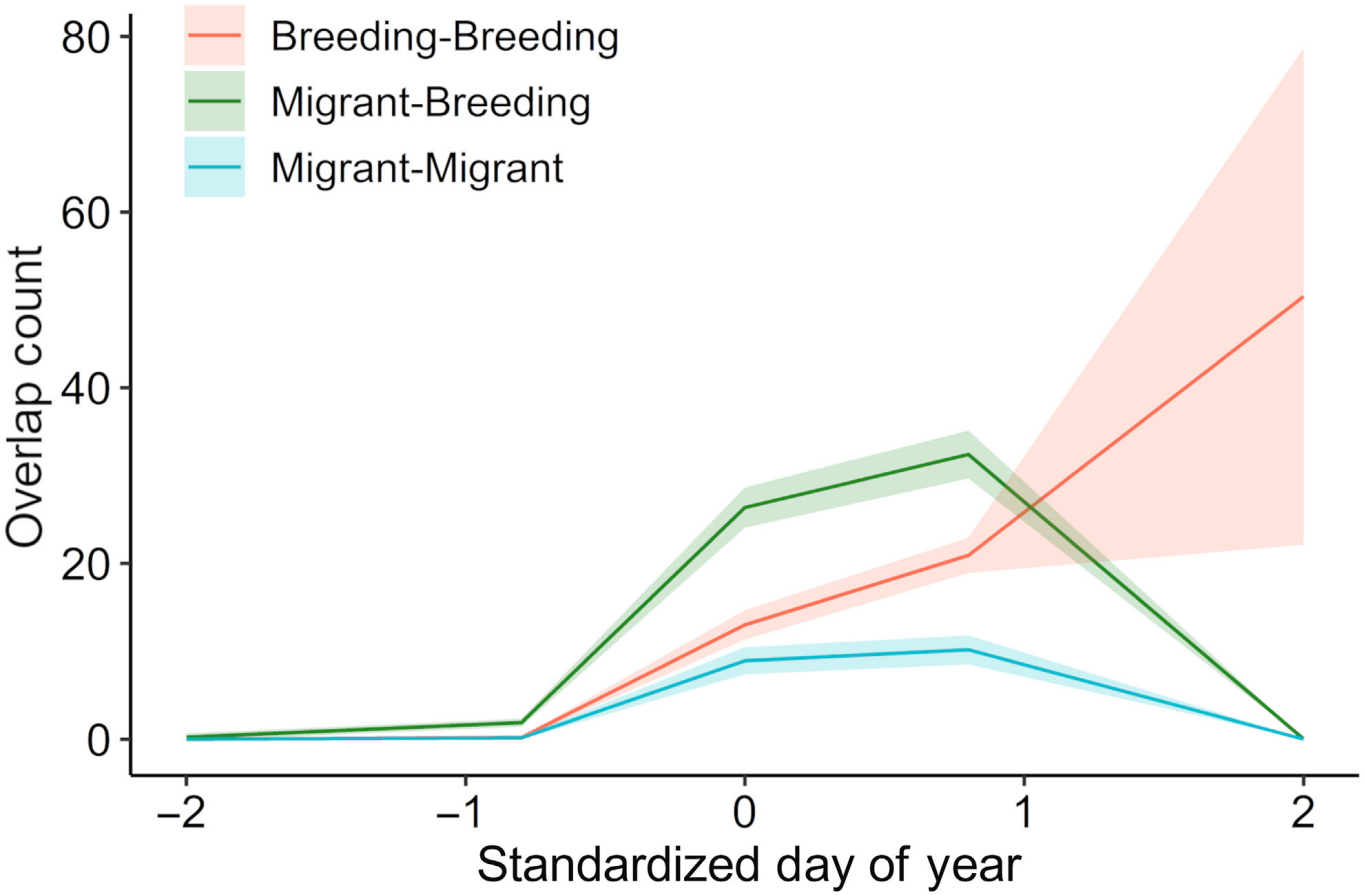
The number of niche overlaps over time varied among migrant‐breeding species pairs (green), breeding‐breeding species pairs (red), and migrant‐migrant pairs (blue). The figure shows estimates from a Poisson generalized linear model for model fit (solid line) and the standard error (shading), with standardized day of year on the *x*‐axis. The results revealed a significant interaction between pair type and the cubic polynomial of day of year (*χ* ^2^ (6,54) = 33.95, *p* < .0001).

Numbers of pairwise overlaps of signaling niches differed among pair types (migrant‐breeding, breeding‐breeding, and migrant‐migrant) and through time. The interaction between pair type and a cubic polynomial of date was significant (GLM, interaction term: *χ* ^2^ (6,54) = 33.95, *p* < .0001; Table A6). During early migration when few species were present in the community, few overlaps occurred across pair types (Figure 6). As both migrant and breeding species arrived, the number of overlaps between migrant and breeding species increased, reaching a peak in the number of overlaps when the community species richness was highest (Figure 6). In contrast, the number of niche overlaps between breeding species increased slowly over time until the whole breeding community was present (Figure 6).

### 3.3 Null model test for dispersion of signaling niches

The null model test revealed that signaling niches of species in the community were overdispersed in signal space. The mean interspecific distance between niches was significantly greater than expected by chance (*Z* = 11.93, *p* < .001). The majority of species pairs (53% of 435 pairs) had greater distances between signaling niches than expected randomly (significantly positive *Z* scores), while 17% of pairs exhibited lesser distances than expected randomly (significantly negative *Z* scores) and 29% of pairs had distances that did not differ from random (nonsignificant *Z* scores).

### 3.4 Phylogenetic relatedness and pairwise proximity in signal space

**FIGURE 7:**
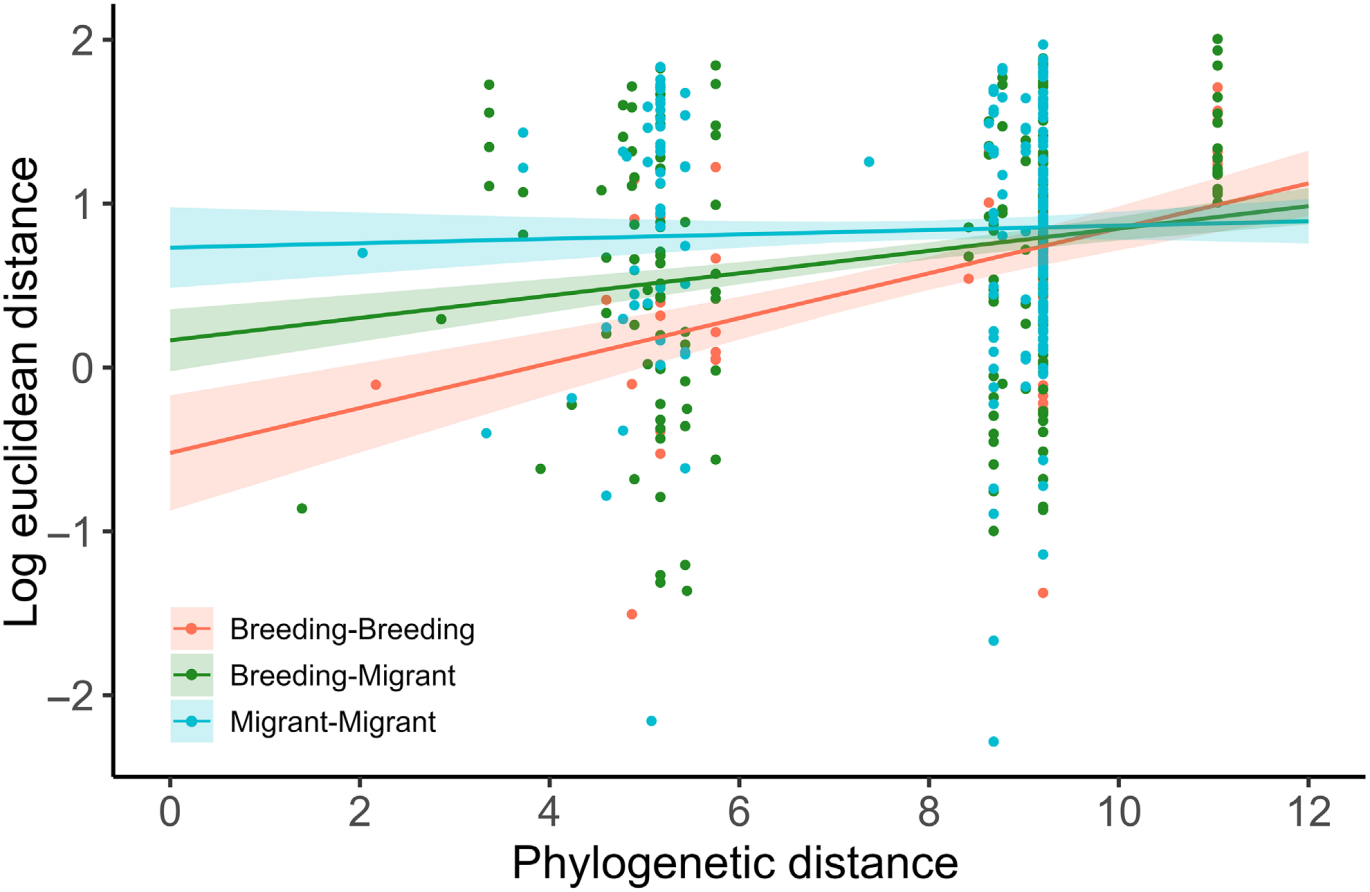
The relationship between phylogenetic distance and log‐transformed Euclidean distance between species' niche centroids in signal space differed among pair types. The figure shows estimates from a linear model for model fit (solid line) and the standard error (shading) with a significant interaction between phylogenetic distance and pair type (*R* ^2^ = .06, *F*_5,429_ = 5.17, *p* < .0001).

The relationship between phylogenetic distance and proximity in signal space differed among pair types (breeding‐breeding, migrant‐breeding, and migrant‐migrant), as the linear model revealed a significant interaction between pair type and phylogenetic distance (*R* ^2^ = .06, *F*_5,429_ = 5.17, *p* < .0001; Figure 7). A strong positive relationship existed between phylogenetic distance and Euclidean distance in signal space between breeding‐breeding pairs: more closely related breeding species shared more similar acoustic traits and were closer to each other in signal space. This relationship was positive, but the slope shallower, for breeding‐migrant pairs compared with breeding pairs, whereas no relationship between phylogenetic distance and Euclidean distance existed between migrant species pairs (Figure 7). In post‐hoc pairwise comparisons, Euclidean distances between niche centroids in signal space differed between breeding‐breeding pairs and migrant‐migrant pairs (*t* ratio = 2.378, *p* = .047), but other comparisons were not significant (Tukey's test: *p* > .05).

## 4 DISCUSSION

We explored the influence of migrant species on niche partitioning in breeding communities by investigating the patterns of overlap in the signal space of migrant and locally breeding warbler species. During the spring migratory period in SW Michigan, 1–17 migrant species sang and co‐occurred with the breeding warbler community, itself composed of 11 species. Migrants were present for much of the early breeding season, extensively overlapping the critical period when males of breeding species sang to attract mates and defend territories. Indeed, acoustic overlap of breeding species by migrant species occurred more commonly than between pairs of breeding species, with the number of overlaps peaking during a 9‐day period when 28 migrant and breeding species co‐occurred. The degree to which breeding species' niches were overlapped varied by species and over time; some species experienced greater overlap from migrants, while others experienced greater overlap from other breeding species. Overall, our results suggest that migratory species act as an unexplored source of acoustic interference within breeding communities they temporarily inhabit en route to their own breeding areas. Singing by migratory species overlaps the signal space occupied by breeding species, with potential impacts on signal detection, discrimination, and evolution.

Species vocalizing at the same time and place encounter communication interference from acoustic competitors, particularly when they share similar acoustic traits (Schmidt et al., 2013). Yet, co‐existing species are predicted to inhabit well‐defined signaling niches (Luther, 2009), which evolve via selection for species‐specific song (Grant & Grant, 2010). In our study of migratory and breeding warblers, some regions of signal space were occupied by few species with little or no overlap, leading to overdispersion in signaling niches in the community. Analysis of dispersion patterns and overlap between species pairs indicated that signaling niches in signal space were largely separated. Indeed, more than 50% of species pairs were overdispersed in signal space. However, some regions were tightly packed with multiple species which had a clumped distribution (17% of species were underdispersed) and high degrees of overlap (Figure 3). Closely related species were generally close together in signal space, yet this varied among pair types with the strongest relationship between phylogenetic and Euclidean distances found in pairs of breeding species, a weaker but positive relationship in breeding‐migrant pairs, and no relationship within migrant pairs (Figure 7). Yet, signaling niches of migrant‐breeding species pairs overlapped two times more than other types of overlap (i.e., breeding‐breeding and migrant‐migrant pairs) and overlap peaked when the community had its highest species richness (Figure 6). Thus, while closely related breeding species were more likely to occupy similar signaling niches than the other pair types, when signaling niche overlaps occurred, migrants more commonly overlapped breeding species than did other breeders.

Breeding species face an influx and continual turnover of migratory species each spring (Kirby et al., 2008). Males of migratory breeding species typically begin singing upon or shortly after arriving on breeding grounds (Slagsvold, 1977), such that early days of breeding are marked by high song rates overall (Spector, 1992). Most warblers in SW Michigan arrive in early May and breed for 3 months during May through July (Kendeigh, 1945, Figure 4), but migration timing differed among the migratory species, which breed north of the study region. Yet even with staggered schedules, multiple migrant species co‐occurred with breeding species over 51 days of the breeding season (Figure 4). During one 9‐day period of migration, 28/30 species (17 migrant, 11 breeding) co‐occurred, nearly tripling the number of warbler species present compared to the locally breeding community alone (Figure 4). This high level of species co‐occurrence was unusual, as migrants typically did not exist in the community simultaneously (≤ five species co‐occurred on 24/51 days), resulting in changing patterns of niche overlap and competition for acoustic space through time. Indeed, differential migration timing may represent an evolved separation of niches among migratory species to partition resource use during the migratory period (Heim et al., 2018; Novcic, 2016) and even closely related species vary greatly in migratory onset, duration, pace, and utilization of stopover sites (Bennett et al., 2017; Helm et al., 2009). Dynamic influxes of migratory singers may alter the availability of signal space for breeding species during most of the critical singing stage, influencing past and present song evolution along the migratory pathway in unexplored ways.

Signaling niches can be partitioned along multiple axes, in addition to the spectral and temporal traits considered here. Individuals of species with signaling niches heavily overlapped by other species could minimize overlap by employing short‐term behavioral adjustments (Wilson et al., 2016), such as staggering singing bouts (Cody & Brown, 1969), singing at different times of day (Luther, 2008), broadcasting songs from different strata of the forest (Jain & Balakrishnan, 2012), or spatially segregating by microhabitats within the same place (Schmidt et al., 2013). Follow‐up studies could investigate real‐time adjustments of singing by breeding species during the migratory period to assess the extent to which breeding species respond to signaling niche overlap by migrants. Additionally, interspecific differences in habitat preferences are likely to reduce niche overlap for breeding species, particularly when considering overlap by other locally breeding species. None of the breeding species in this community overlap entirely in habitat requirements (Chartier et al., 2011) and up to 5 of the 11 breeding species are likely to co‐occur in the same habitat type. Thus, although some breeding species, such as cerulean warblers, were heavily overlapped in signal space by other locally breeding species, they are unlikely to breed in the same habitat; in nature, spatial segregation by habitat likely minimizes acoustic interference for some breeding species (Chitnis et al., 2020). However, migrants are typically habitat generalists, using a broader range of habitats on stopovers than they would during the breeding or overwintering seasons (Chernetsov, 2006; Parnell, 1969). Whereas breeding species may breed in distinct habitats, thereby minimizing overlap, singing migrant species may act as an important and variable source of acoustic interference for breeding species, highlighting the importance of considering migratory species in studies of resource partitioning in breeding communities. Additional study considering spatially explicit patterns of overlap of breeding species by both breeding and migrant species would provide insight into additional processes minimizing acoustic interference.

Migratory species may influence singing and signaling niches of individual breeding species in communities to varying degrees. Some breeding species in the SW Michigan community experienced a high proportion of niche overlap from migrants, especially at the peak of migration (Figure A3), such that singing migrants may constrain the availability of signal space for these species much more than co‐occurring breeding species. The mechanisms used by breeding species to avoid song overlap with transient species, much like the impact of migratory species on resource competition more generally, remains largely unexplored. Evidence from community niche partitioning at overwintering and stopover sites suggests that rather than maintain niches overlapped by migrants, resident species plastically shift their niches to alleviate competition during periods when migrants are abundant (Jedlicka et al., 2006; Waide, 1981). When coexisting with migrants, resident species display ecological shifts in foraging niches (Jedlicka et al., 2006), minimal overlap in diet (Poulin & Lefebvre, 1996), spatial segregation via microhabitat selection (Bensusan et al., 2011; Ortega‐Álvarez et al., 2020), and increases in territorial and calling behavior (Bensusan et al., 2011). Similarly, breeding species that share tightly packed areas of signal space with migrants (Figure 3) could partition the acoustic resource via spatial and/or temporal avoidance (Planqué & Slabbekoorn, 2008). Further study is needed to explore the strategies breeding species may use to avoid signal overlap when migrants are present and singing.

By investigating patterns of signaling niche overlap in a community of breeding and migrant warblers over the course of migration, we showed that migratory species may act as important, but overlooked, drivers of signaling niche partitioning and signal evolution in breeding bird communities. Singing migrants were present in the community for two‐thirds of the breeding season, overlapping the signal space of breeding species during their critical period of mate attraction and territorial defense. Migrants overlapped the songs of breeding species to varying extents such that not all species were affected in the same way. Acoustic competition among co‐occurring species shapes signal structure over evolutionary time to partition signal space and promote intraspecific communication (Grant & Grant, 2010). By coexisting annually with breeding species during a critical life history stage, singing migrants may act as additional acoustic competitors, imposing constraints on song detection and selection for partitioning in signal space. Additional work exploring the prevalence of migrant singing and whether breeding species behaviorally shift signaling niches when migrants are present will be critical for understanding the impacts of migratory species on partitioning of the acoustic resource, and signal evolution more broadly. Our research contributes to a growing body of evidence that including migratory species in community analyses broadens our understanding of the evolutionary and ecological factors shaping ecological systems.

## AUTHOR CONTRIBUTIONS

**Joanna M. Sblendorio:** Conceptualization (equal); data curation (lead); formal analysis (equal); funding acquisition (equal); investigation (lead); methodology (equal); project administration (lead); software (lead); validation (equal); visualization (lead); writing – original draft (equal); writing – review and editing (equal). **Maarten J. Vonhof:** Conceptualization (equal); methodology (equal); resources (supporting); writing – review and editing (supporting). **Sharon A. Gill:** Conceptualization (equal); data curation (supporting); formal analysis (equal); funding acquisition (equal); investigation (supporting); methodology (equal); project administration (equal); resources (lead); software (supporting); supervision (lead); validation (equal); visualization (supporting); writing – original draft (equal); writing – review and editing (equal).

## FUNDING INFORMATION

This work was supported by funding from Western Michigan University (to S.A.G.) and the Animal Behavior Society (Student Research Grant to J.M.S.).

## CONFLICT OF INTEREST STATEMENT

The authors declare that they have no competing interests.

## Data Availability

The data supporting this study are openly available at the Dryad Digital Repository (doi: 10.5061/dryad.2jm63xswx). The eBird basic dataset is available for download at: ebird.org/data/download.

## APPENDIX 1

**TABLE A1:** A list of the 30 warbler species included in this study, showing the species‐specific band pass frequency filters used on spectrograms in Avisoft‐SASLab Pro v5.2.13 (Specht, 2002) prior to final song data extraction.

Species	Band pass filter (kHz)
American Redstart	2.5–10.0
Bay‐breasted Warbler	3.9–10.0
Black‐and‐white Warbler	3.0–11.0
Blackburnian Warbler	2.7–12.5
Blackpoll Warbler	6.8–11.3
Black‐throated Blue Warbler	2.6–7.7
Black‐throated Green Warbler	3.0–8.5
Blue‐winged Warbler	3.0–11.0
Canada Warbler	1.9–10
Cape May Warbler	3.4–10.3
Cerulean Warbler	2.0–8.5
Chestnut‐sided Warbler	2.0–9.5
Common Yellowthroat	1.5–8.0
Connecticut Warbler	1.0–8.8
Golden‐winged Warbler	3.0–11.0
Hooded Warbler	1.5–8.0
Magnolia Warbler	2.1–8.5
Mourning Warbler	1.5–8.6
Nashville Warbler	2.0–10.5
Northern Parula	2.6–9.0
Northern Waterthrush	1.5–9.0
Ovenbird	1.5–10.5
Palm Warbler	2.3–8.5
Pine Warbler	2.5–6.5
Prairie Warbler	3.0–8.0
Prothonotary Warbler	2.2–10.0
Tennessee Warbler	2.0–11.5
Wilson's Warbler	2.0–9.7
Yellow Warbler	2.0–10.0
Yellow‐rumped Warbler	2.3–8.0

**TABLE A2:** Loadings of song traits of 30 warbler species on the two principal components (PCs) with eigenvalues >1.

	PC1	PC2
Eigenvalue	1.5714	1.5006
Variance explained	41.15%	37.53%

Song variable	Loadings	Loadings
Duration (s)	0.099	**−0.554**
Peak frequency (Hz)	**0.605**	0.120
Min frequency (Hz)	**0.489**	0.348
Max Freq (Hz)	**0.597**	−0.143
Bandwidth (Hz)	0.165	**−0.505**
Number of notes	0.037	**−0.531**

*Note*: Variables with high loadings on each PC axis (>0.4 or <−0.4) are shown in bold.

**TABLE A3:** Comparison of candidate binomial generalized linear mixed models explaining change in the proportion overlap of breeding species' niches by migrants versus other breeding species during migration (*n* = 262).

Candidate models	Residual deviance	Deviance change	*p* for *χ* ^2^
Type + DOY + Type * DOY	280.19		
Type + DOY^2^ + Type * DOY^2^	220.26	−59.93	.00
**Type + DOY** ^**3**^ **+ Type * DOY** ^**3**^	**217.12**	**−3.13**	**.21**

*Note*: Predictors included overlap type (breeding, migrant), day of year (DOY) or polynomial of DOY, and the interaction between overlap type and DOY or the polynomial of DOY. All models included species as a random effect. Model selection revealed that the quadratic model was the best fit, but model assumption testing showed inextricable issues with overdispersion. Therefore, we selected the cubic model (shown in bold).

**TABLE A4:** Comparison of candidate Poisson generalized linear models explaining change in pairwise overlap counts (*n* = 66) by pair type during spring migration.

Candidate models	Residual deviance (df)	Deviance change	*p* for *χ* ^2^
Type + DOY + Type * DOY	646.86 (60)		
Type + DOY^2^ + Type * DOY^2^	64.33 (57)	−582.54	.00
**Type + DOY** ^**3**^ **+ Type * DOY** ^**3**^	**33.95 (54)**	−**30.37**	**.00**

*Note*: A polynomial term was needed in the model to account for typical patterns of migration. Predictors included pair type (migrant‐migrant, migrant‐breeding, breeding‐breeding), day of year (DOY) or polynomial of DOY, and the interaction between pair type and DOY or the polynomial of DOY. Model selection revealed that the model containing the cubic term was the best fit (shown in bold) and reduced deviance the most.

**TABLE A5:** A binomial generalized linear mixed model with a logit link function representing change in the proportion niche overlap experienced by breeding species from migrants and other breeding species over the spring migratory period (*n* = 262 observations).

Predictors	Overlap proportion				
	Estimate	SE	CI	*z*	*p*
(breeding)	−0.64	0.57	−1.76 to 0.48	−1.12	.265
Overlap Type [migrant]	−1.56	0.54	−2.61 to −0.50	−2.89	**.004**
DOY, 3 [1st degree]	4.40	4.36	−4.14 to 12.94	1.01	.313
DOY, 3 [2nd degree]	−7.07	4.85	−16.58 to 2.43	−1.46	.145
DOY, 3 [3rd degree]	6.84	5.01	−2.98 to 16.65	1.36	.172
Type [migrant] * DOY, 3 [1st degree]	−19.28	16.93	−52.46 to 13.91	−1.14	.255
Type [migrant] * DOY, 3 [2nd degree]	−42.70	12.53	−67.26 to −18.14	−3.41	**<.001**
Type [migrant] * DOY, 3 [3rd degree]	−21.90	15.58	−52.43 to 8.63	−1.41	.160

Random effects	
*σ* ^2^	3.29
*τ*_00 Species_	2.67
ICC	0.45
*N*_Species_	10

*Note*: Our model predictors are overlap type (breeding, migrant), the cubic polynomial of day of year (DOY), and their interactions, with species as a random effect. Log‐odds estimates, 95% confidence intervals (CIs), *z*‐statistics, and *p*‐values are shown. All significant relationships (*p* < .05) are in bold.

**TABLE A6:** A Poisson generalized linear model with a log link function representing change in the number of pairwise overlap counts (*n* = 63 observations) as a function of pair type and cubic day of year (DOY) over the spring migratory period.

Predictors	Estimate	Overlap count			
		SE	CI	*z*	*p*
(breeding‐breeding)	−0.34	0.61	−1.66 to 0.72	−0.56	.577
Type [migrant‐breeding]	1.74	0.63	0.61 to 3.09	2.74	**.006**
Type [migrant‐migrant]	−0.50	1.06	−2.90 to 1.34	−0.48	.635
DOY, 3 [1st degree]	33.61	6.42	22.35 to 47.44	5.23	**<.001**
DOY, 3 [2nd degree]	−21.04	4.56	−30.73 to −12.91	−4.61	**<.001**
DOY, 3 [3rd degree]	6.65	1.92	3.09 to 10.61	3.46	**.001**
Type [migrant‐breeding] * DOY, 3 [1st degree]	−27.89	6.82	−42.33 to −15.50	−4.09	**<.001**
Type [migrant‐migrant] * DOY, 3 [1st degree]	−18.17	13.11	−40.02 to 10.71	−1.39	.166
Type [migrant‐breeding] * DOY, 3 [2nd degree]	7.46	4.79	−1.27 to 17.50	1.56	.119
Type [migrant‐migrant] * DOY, 3 [2nd degree]	−0.91	8.12	−19.02 to 13.06	−0.11	.911
Type [migrant‐breeding] * DOY, 3 [3rd degree]	−12.50	2.35	−17.21 to −7.96	−5.31	**<.001**
Type [migrant‐migrant] * DOY, 3 [3rd degree]	−10.53	5.27	−19.50 to 0.32	−2.00	**.046**

*Note*: The predictors in our model are pair type (migrant‐migrant, migrant‐breeding, breeding‐breeding), the cubic polynomial of DOY, and the interaction between pair type and the cubic polynomial of DOY. Log‐mean estimates, 95% confidence intervals (CIs), *z*‐statistics, and *p*‐values are shown. All significant relationships (*p* < .05) are bolded.
